# Geographic distribution and genetic diversity of Whitewater Arroyo virus in the southwestern United States.

**DOI:** 10.3201/eid0703.010306

**Published:** 2001

**Authors:** C F Fulhorst, R N Charrel, S C Weaver, T G Ksiazek, R D Bradley, M L Milazzo, R B Tesh, M D Bowen

**Affiliations:** University of Texas Medical Branch, Galveston, Texas 77555-0609, USA. cfulhorst@utmb.edu

## Abstract

The purpose of this study was to extend our knowledge of the geographic distribution and genetic diversity of the arenavirus(es) associated with Neotoma species (woodrats) in the southwestern United States. Infectious arenavirus was recovered from 14 (3.3%) of 425 woodrats. The virus-positive species included N. albigula in New Mexico and Oklahoma, N. cinerea in Utah, N. mexicana in New Mexico and Utah, and N. micropus in Texas. Analyses of viral nucleocapsid protein gene sequence data indicated that all the isolates were strains of the Whitewater Arroyo virus, an arenavirus previously known only from northwestern New Mexico. Analyses of the sequence data also indicated that there can be substantial genetic diversity among strains of Whitewater Arroyo virus from conspecific woodrats collected from different localities and substantial genetic diversity among strains from different woodrat species collected from the same locality.


### Geographic Distribution and Genetic Diversity of Whitewater Arroyo Virus in the Southwestern United States

**Charles F. Fulhorst,* Remi N. Charrel,*† Scott C. Weaver,* Thomas G. Ksiazek,‡ Robert D. Bradley,§ Mary L. Milazzo,* Robert B. Tesh,* and Michael D. Bowen‡**
*University of Texas Medical Branch, Galveston, Texas, USA; †Faculté de Médecine, Marseille, France; ‡Centers for Disease Control and Prevention, Atlanta, Georgia, USA; and §Texas Tech University, Lubbock, Texas, USA

The purpose of this study was to extend our knowledge of the geographic distribution and genetic diversity of the arenavirus(es) associated with *Neotoma* species (woodrats) in the southwestern United States. Infectious arenavirus was recovered from 14 (3.3%) of 425 woodrats. The virus-positive species included *N. albigula* in New Mexico and Oklahoma, *N. cinerea* in Utah, *N. mexicana* in New Mexico and Utah, and *N. micropus* in Texas. Analyses of viral nucleocapsid protein gene sequence data indicated that all the isolates were strains of the Whitewater Arroyo virus, an arenavirus previously known only from northwestern New Mexico. Analyses of the sequence data also indicated that there can be substantial genetic diversity among strains of Whitewater Arroyo virus from conspecific woodrats collected from different localities and substantial genetic diversity among strains from different woodrat species collected from the same locality.

The virus family *Arenaviridae* comprises two serocomplexes. The lymphocytic choriomeningitis-Lassa (Old World) complex includes lymphocytic choriomeningitis (LCM), Lassa, Mopeia, Mobala, and Ippy viruses. The Tacaribe (New World) complex includes Tamiami (TAM), Whitewater Arroyo (WWA), Pichindé (PIC), Amapari, Flexal, Guanarito, Junin, Latino, Machupo, Oliveros, Parana, Pirital, Sabiá, and Tacaribe viruses.

The arenaviruses have bipartite, single-stranded RNA genomes (1). The large (L) genomic segment encodes the viral RNA-dependent RNA polymerase and a zinc-binding protein. The small (S) genomic segment encodes the nucleocapsid (N) protein and glycoprotein precursor. The most comprehensive knowledge of the phylogeny of the family *Arenaviridae* is based on a fragment of the N protein gene (2-4).

Six arenaviruses are known to cause severe disease in humans. LCM virus is an agent of acute central nervous system disease (5) and congenital malformations (6). Lassa, Junin, Machupo, Guanarito, and Sabiá viruses are etiologic agents of hemorrhagic fever in western Africa, Argentina, Bolivia, Venezuela, and Brazil, respectively (7).

The arenaviruses known to occur in North America are LCM, TAM, and WWA. LCM virus was introduced into the Americas along with its principal rodent host, *Mus musculus* (house mouse) (8). TAM virus is known only from *Sigmodon hispidus* (hispid cotton rat) in southern Florida (9-11). WWA virus was originally recovered from *Neotoma albigula* (white-throated woodrat) collected from northwestern New Mexico (12).

Address for correspondence: Charles F. Fulhorst, Department of Pathology, Route 0609, University of Texas Medical Branch, Galveston, Texas 77555-0609, USA; fax: 409-747-2415; e-mail: cfulhors@utmb.edu

In a recent study (13), antibody to an arenavirus was found in five *Neotoma* species in the southwestern United States: *N. albigula* in Arizona, Colorado, and New Mexico; *N. stephensi* (Stephen's woodrat) in Arizona and New Mexico; *N. mexicana* (Mexican woodrat) in Arizona and Utah; and *N. fuscipes* (dusky-footed woodrat) and *N. lepida* (desert woodrat) in California. The purpose of the present study was to extend our knowledge of the geographic distribution and genetic diversity of the arenavirus(es) associated with *Neotoma* rodents in the southwestern United States.

### Materials and Methods

All work with rodent tissues and infectious arenavirus was performed in a biosafety level 3 laboratory at the Centers for Disease Control and Prevention (Atlanta, GA) or University of Texas Medical Branch, Galveston.

### Rodent Tissues

Five hundred sixty-six tissue specimens (74 spleen, 225 liver, and 267 kidney) from 425 woodrats were tested for infectious arenavirus. The specimens were from the Museum of Texas Tech University (Lubbock, TX) or Museum of Southwestern Biology (University of New Mexico, Albuquerque, NM). The specimens from the Museum of Southwestern Biology were chosen to represent localities in which antibody to an arenavirus was found in one or more *Neotoma* species in a previous study (13).

### Virus Assay

Tissue specimens were tested for infectious arenavirus as described previously (12). Briefly, 0.2 mL of a 10% w/v crude tissue homogenate was inoculated onto a monolayer of Vero E6 cells in a 25-$cm^2$ plastic culture flask (Corning, Inc.,

Corning, NY). The inoculum was incubated on the cell monolayer at 37°C for 60 minutes; then the monolayer was overlaid with 7.0 mL of a minimum essential medium containing Earle's salts, 1.5 mg/mL sodium bicarbonate, 2% v/v heat-inactivated (56°C for 30 minutes) fetal bovine serum, 0.29 mg/mL L-glutamine, 100 U/mL penicillin G, 100 µg/mL streptomycin sulfate, and 100 U/mL nystatin. The cell culture was maintained at 37°C in a humidified atmosphere of 5% $CO_2$ in air for 13 days. Half the culture medium was replaced with fresh maintenance medium on day 6 or 7 after inoculation. Cells were scraped from the monolayer on day 13 after inoculation and coated onto 12-well glass microscope slides (Cel-Line Associates, Inc., Newfield, NJ). The cell spots were air-dried, fixed in cold acetone, and then tested for arenaviral antigen by using an indirect fluorescent antibody test (12). In that test, cell spots were stained with a hyperimmune mouse ascitic fluid prepared against the WWA virus prototype strain AV 9310135, and mouse immunoglobulin G (IgG) bound to cell-associated arenaviral antigen was detected by using a goat anti-mouse IgG fluorescein isothiocyanate conjugate (Cappel Laboratories, West Chester, PA).

### Genetic Characterization of Viral Isolates

The nucleotide sequence of a fragment of the N protein gene of each of 12 isolates was determined. Four of the 12 isolates were from the spleens and kidneys of two animals, rodents 62425 and 62439 (Table 1). Total RNA was extracted from monolayers of infected Vero E6 cells by using TRIzol Reagent (Life Technologies, Inc., Grand Island, NY). Reverse transcription of RNA from isolates AV 96010149, AV 96010151, AV 96010025, and AV 96010024 was carried out by using Superscript II RTase (Life Technologies) in conjunction with oligonucleotide ARE-3'END (14). This oligonucleotide apparently is complementary to the 19-nt fragment at the 3' terminus of the S genomic segment of all arenaviruses. Polymerase chain reaction (PCR) amplification of the first-strand cDNA was carried out by using *Taq* DNA polymerase (Promega Corp., Madison, WI) in conjunction with oligonucleotides 1010C and NW1696R (2-3), which flank a 616-nt region of the N protein gene of WWA virus prototype strain AV 9310135 (12). Reverse transcription and PCR (RT-PCR) amplification of a fragment of the N protein gene of each of the eight other isolates was carried out by using the Access RT-PCR Kit (Promega Corp.) in conjunction with oligonucleotides AVNP1 (5'-CCCTTCTTYTTNYTCTTRATGACTA-3') and AVNP2 (5'-GGKAGRGCNTGGGAYAACAC-3'). AVNP1 and AVNP2 flank a 518-nt region in the fragment of the WWA virus N protein gene that is amplified by using oligonucleotides 1010C and NW1696R. They were designed based on N protein gene sequence data for the WWA virus prototype strain AV 9310135 (GenBank Accession No. U52180), WWA virus strains AV 96010149, AV 96010151, AV 96010025, and AV 96010024, TAM virus strain W-10777 (U43690), and PIC virus strain An 3739 (K02734). Size separation of PCR products was done by agarose gel electrophoresis; the products of the expected size were purified from gel slices by using QIAquick Gel Extraction Kit (Qiagen, Inc., Valencia, CA). One strand of each 1010C-NW1696R PCR product was sequenced directly by using the dye termination cycle sequencing technique (Applied Biosystems, Inc., Foster City, CA) in conjunction with oligonucleotide 1010C. The sequence of the other (i.e., complementary) strand of each of these products was determined by cloning the PCR product in the TA cloning vector PCRII (Invitrogen Corp., Carlsbad, CA) and then using a plasmid-specific oligonucleotide (M13) to initiate the cycle sequencing reaction. Both strands of the AVNP1-AVNP2 PCR products were sequenced directly by using the same oligonucleotides that were used to prime the RT-PCR, i.e., AVNP1 and AVNP2. The 12 nucleotide sequences generated in this study were deposited with the GenBank nucleotide sequence database under Accession Nos. AY012710-AY012721.

### Data Analysis

The analyses of nucleotide sequence data were restricted to the 518-nt fragment of the WWA virus N protein gene that is flanked by oligonucleotides AVNP1 and AVNP2. The GenBank database sequences included in the analyses were Accession Nos. U52180 (WWA virus, strain AV 9310135), U43690 (TAM, W-10777), K02734 (PIC, An 3739), U43689 (Parana, 12056), U43687 (Flexal, BeAn 293022), U62561 (Pirital, VAV-488), U43688 (Latino, 10924), U34248 (Oliveros, 3229-1), U70802 (Junin, XJ), X62616 (Machupo, AA288-77), U43686 (Guanarito, INH-95551), U41071 (Sabiá, SPH 114202), U43685 (Amapari, BeAn 70563), M20304 (Tacaribe, TRVL 11573), M20869 (LCM, Armstrong), and U80004 (Lassa, LP). The computer software package

Table 1. Recovery of infectious arenavirus from tissues of virus-positive woodrats (*Neotoma* species)

| | | Date | Collected from | | Virus (strain) recovered from[a] | | |
|---|---|---|---|---|---|---|---|
| Rodent | Species | collected | County | State | Spleen | Kidney | Liver |
| 1627 | *N. albigula* | 07/15/93 | McKinley | NM | AV 9310041 | **AV 9310135** | nt |
| 1626 | *N. albigula* | 07/15/93 | McKinley | NM | nt | AV 9310040 | nt |
| 62415 | *N. mexicana* | 09/24/94 | Socorro | NM | nt | AV 96010149 | nt |
| 62425 | *N. mexicana* | 09/24/94 | Socorro | NM | nt | AV 96010151 | AV 98360019 |
| 62439 | *N. mexicana* | 09/24/94 | Socorro | NM | nt | AV 96010154 | AV 98360020 |
| 28731 | *N. albigula* | 10/12/85 | Cimarron | OK | nt | AV 98490013 | Negative |
| 28742 | *N. albigula* | 10/12/85 | Cimarron | OK | nt | AV 97130039 | TVP-6038 |
| 84648 | *N. micropus* | 07/18/99 | Dimmit | TX | Negative | AV A0400098 | nt |
| 84703 | *N. micropus* | 07/19/99 | Dimmit | TX | AV A0400337 | AV A0400135 | nt |
| 84708 | *N. micropus* | 07/19/99 | Dimmit | TX | nt | AV A0400140 | nt |
| 84761 | *N. micropus* | 07/18/99 | Dimmit | TX | AV A0400373 | AV A0400174 | nt |
| 84816 | *N. micropus* | 07/20/99 | La Salle | TX | AV A0400412 | AV A0400212 | nt |
| 36287 | *N. cinerea* | 07/06/94 | San Juan | UT | nt | AV 96010025 | AV 96010206 |
| 36282 | *N. mexicana* | 07/05/94 | San Juan | UT | nt | AV 96010024 | AV 96010205 |

[a]nt = not tested. The WWA virus prototype strain is bolded. Isolates (strains) included in the (phylo-) genetic analyses are underlined.

CLUSTAL W1.7 (15) was used to construct an alignment of the predicted amino acid sequences, and the computer program TransAlign (16) was used to generate a multiple nucleotide sequence alignment from the amino acid sequence alignment. Pairwise genetic distances were computed by using the p distance model as implemented in the computer program MEGA, version 1.02 (17). Percent sequence identities were calculated by subtracting the genetic distances from 1.0 and multiplying by 100. Phylogenetic analysis was carried out on the multiple amino acid sequence alignment by using the neighbor-joining method (gamma model, alpha = 2) as implemented in MEGA, version 1.02. Bootstrap support (18) for the results of the phylogenetic analysis was based on 500 pseudoreplicate datasets generated from the original multiple amino acid sequence alignment.

### Results

### Viral Isolates

Twenty-three arenaviral isolates were recovered from tissues of 14 (3.3%) of 425 *Neotoma* rodents (Table 1). The 23 isolates included three WWA virus strains (AV 9310135, AV 9310041, and AV 9310040) that were reported previously (12).

The virus-positive animals included two *N. albigula* from McKinley County, northwestern New Mexico; two *N. albigula* from Cimarron County, western Oklahoma; three *N. mexicana* from Socorro County, central New Mexico; five *N. micropus* from the Chaparral Wildlife Management Area (Dimmit and La Salle counties), southern Texas; and one *N. mexicana* and one *N. cinerea* from San Juan County, southeastern Utah (Table 2, Figure 1). The virus-positive animals from McKinley County were two (50%) of four woodrats (all *N. albigula*) collected on July 15, 1993, from Whitewater Arroyo. The positive *N. albigula* from Cimarron County were two (22.2%) of nine woodrats (seven *N. albigula* and two *N. mexicana*) collected on October 12, 1985, from a site near Kenton. The positive *N. mexicana* from Socorro County were three (42.9%) of seven woodrats (all *N. mexicana*) collected on September 24, 1994, from a site in the Magdalena Mountains. The positive *N. micropus* from Dimmit County were 4 (13.8%) of 29 woodrats (all *N. micropus*) collected in a 3-day period (July 17 through July 19, 1999) from the western region of the Chaparral Wildlife Management Area. The positive *N. micropus* from La Salle County was one (25.0%) of four woodrats (all *N. micropus*) collected on July 20, 1999, from the eastern region of the Chaparral Wildlife Management Area. The positive *N. mexicana* and *N. cinerea* from San Juan County were 2 (12.5%) of 16 woodrats (11 *N. mexicana*, 2 *N. cinerea*, and 3 *N. albigula*) collected in an 8-day period (June 29 through July 6, 1994) from Natural Bridges National Monument. Information from the Museum of Southwestern Biology indicated that the positive *N. mexicana* and *N. cinerea* were collected from different sites in Natural Bridges National Monument.

Table 2. Results of virus isolation attempts on tissues from 425 woodrats

| County[b] | State | *Neotoma* species[a] Nalb | Ncin | Nflo | Nme | Nmic | Nste | Total |
|---|---|---|---|---|---|---|---|---|
| Apache (1) | AZ | - | - | - | 0/6 | - | 0/5 | 0/11 |
| Cochise (2) | AZ | 0/31 | - | - | - | - | - | 0/31 |
| Coconino (2) | AZ | 0/6 | - | - | - | - | 0/3 | 0/9 |
| Maricopa (1) | AZ | 0/23 | - | - | - | - | - | 0/23 |
| Navajo (1) | AZ | 0/29 | 0/1 | - | 0/5 | - | 0/7 | 0/42 |
| Yavapai (2) | AZ | 0/5 | - | - | 0/2 | - | - | 0/7 |
| McKinley (3) | NM | 2/16 | - | - | - | - | - | 2/16 |
| Otero (9) | NM | 0/33 | - | - | 0/9 | 0/35 | - | 0/77 |
| Socorro (3) | NM | 0/31 | - | - | 3/10 | 0/1 | - | 3/42 |
| Cimarron (4) | OK | 2/11 | - | - | 0/5 | - | - | 2/16 |
| Major (4) | OK | - | - | 0/45 | - | 0/38 | - | 0/83 |
| McIntosh (2) | OK | - | - | 0/12 | - | - | - | 0/12 |
| Pottawatomie (2) | OK | - | - | 0/7 | - | - | - | 0/7 |
| Dimmit (1) | TX | - | - | - | - | 4/29 | - | 4/29 |
| La Salle (1) | TX | - | - | - | - | 1/4 | - | 1/4 |
| San Juan (8) | UT | 0/3 | 1/2 | - | 1/11 | - | - | 2/16 |
| Total | | 4/188 | 1/3 | 0/64 | 4/48 | 5/107 | 0/15 | 14/425 |

[a]Nalb = *Neotoma albigula,* Ncin = *N. cinerea*, Nflo = *N. floridana*, Nmex = *N. mexicana*, Nmic = *N. micropus*, Nste = *N. stephensi.* Values are the number positive/number tested; "-" = none tested.
[b]Number in parentheses indicates the number of sites sampled in the county.

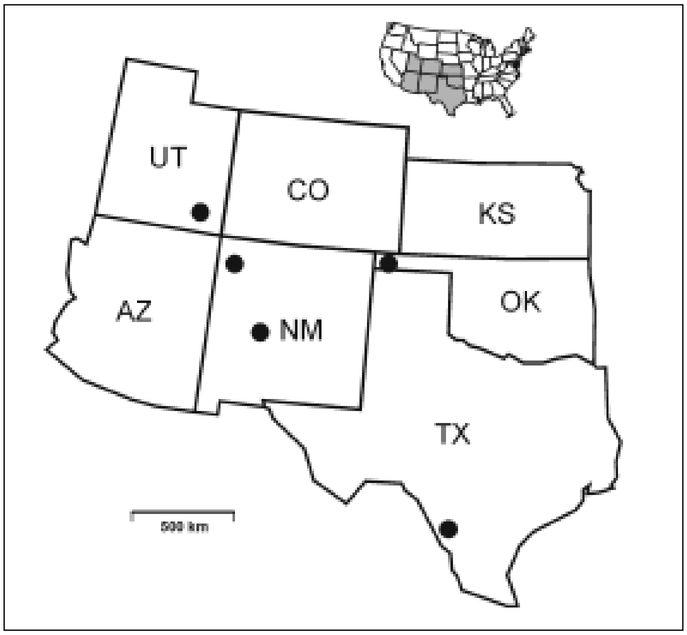

Figure 1. Locations of 14 arenavirus-positive *Neotoma* rodent collections. San Juan County, southeastern Utah = *N. cinerea* and *N. mexicana* (one virus-positive animal each species); Cimarron County, western Oklahoma = *N. albigula* (2); McKinley County, northwestern New Mexico = *N. albigula* (2); Socorro County, central New Mexico = *N. mexicana* (3); Dimmit and La Salle counties (Chaparral Wildlife Management Area), southern Texas = *N. micropus* (5). The map inset shows the location of study area.

The nucleotide sequences of the isolates from rodent 62425 (one isolate each from kidney and liver; strains AV 96010151 and AV 98360019, respectively) were identical. In contrast, the nucleotide sequences of the isolates from rodent 62439 (again, one isolate each from kidney and liver; strains AV 96010154 and AV 98360020, respectively) were 99.6% identical. Further study is needed to determine whether the differences between the isolates from rodent 62439 represent the coexistence of multiple virus genotypes (alleles) in the same rodent. An alternative explanation is that the sequence differences are the result of adaptation of the isolates to growth in cultured (Vero E6) cells or manipulation of viral nucleic acid extracted from cultured cells.

Nucleotide and amino acid sequence identities among WWA virus prototype strain AV 9310135 and 12 other isolates from *Neotoma* rodents ranged from 74.7% to 100.0% and 84.9% to 100.0%, respectively (Table 3). When compared with other arenaviruses, the isolates from the *Neotoma* rodents exhibited 69.9% to 73.7% nucleotide sequence identity with TAM virus, 61.0% to 63.3% identity with PIC virus, and less than 62.0% sequence identity with all other arenaviruses.

Phylogenetic analysis of N protein amino acid sequence data indicated that isolates from *Neotoma* rodents represent a phylogenetic lineage (viral species) that is in a sister relationship to the lineage represented by TAM virus (Figure 2). We concluded that all isolates recovered from the *Neotoma* rodents were strains of WWA virus.

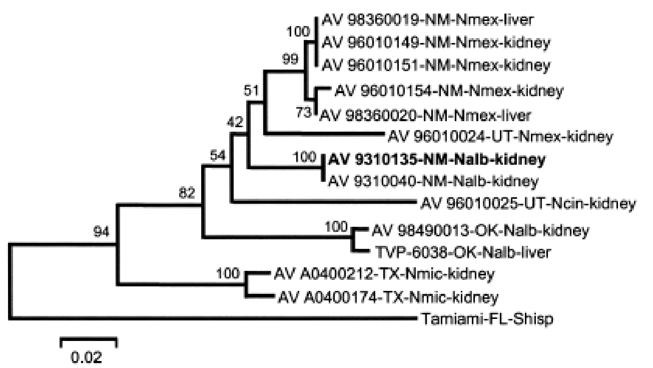

Figure 2. Phylogeny of the North American arenaviruses based on a neighbor-joining analysis of nucleocapsid protein amino acid sequence data. Distances and groupings were determined by using the gamma distance algorithm (alpha = 2). Branch lengths are proportional to the gamma distance between amino acid sequences. Numbers indicate the percentage of 500 bootstrap replicates that supported each labeled interior branch. The WWA virus prototype strain AV 9310135 is in bold type. Nmex = *Neotoma mexicana*, Nalb = *N. albigula*, Ncin = *N. cinerea*, Nmic = *N. micropus*, and Shisp = *Sigmodon hispidus*.

### Discussion

Before the present study, WWA virus was known only from *N. albigula* in northwestern New Mexico (12). The present work provides unequivocal evidence that the virus also is naturally associated with *N. cinerea*, *N. mexicana*, and *N. micropus*, and that it occurs in Utah, central New Mexico, Oklahoma, and Texas. The recovery of WWA virus strains AV 98490013 and TVP-6083 from *N. albigula* is the first evidence that a Tacaribe complex virus occurs in Oklahoma. Likewise, the recovery of strains AV A0400174 and AV A0400212 from woodrats collected from southern Texas (Chaparral Wildlife Management Area) is the first evidence that *N. micropus* is naturally associated with a Tacaribe complex virus and that WWA virus occurs in Texas.

In a previous study (13), antibody to an arenavirus was found in *N. fuscipes* and *N. lepida* in southern California; *N. albigula*, *N. mexicana*, and *N. stephensi* in Arizona; and *N. albigula* in southwestern Colorado. Although the results of the present study indicate that WWA virus is geographically widely distributed in association with *Neotoma* rodents, further work is needed to determine whether the arenavirus associated with *Neotoma* rodents in California, Arizona, and Colorado is in fact WWA virus.

The results of the present study indicate that there can be substantial genetic heterogeneity among strains of WWA virus from different woodrat species from the same locality and among strains from conspecific woodrats collected from different localities. For example, nucleotide sequence identity between the strains recovered from *N. mexicana* and *N. cinerea* from Natural Bridges National Monument (San Juan County, Utah; strains AV 96010024 and AV 96010025, respectively) was 82.8%, and nucleotide sequence identity between strain AV 96010024 and the three strains recovered from *N. mexicana* collected from the Magdalena Mountains (Socorro County, New Mexico; strains AV 96010149, AV 96010151, and AV 96010154) was from 85.1% to 85.5%. In contrast, nucleotide sequence identity in strains recovered from conspecific rodents collected from the same locality (e.g., strains AV 9310135 and AV 9310040 from *N. albigula* from Whitewater Arroyo, and strains AV A0400174 and AV

Table 3. Nucleotide and amino acid sequence identities among 13 arenavirus isolates recovered from 11 woodrats and Tamiami virus[a]

| | | Virus or strain | | | | | | | | | | | | | |
|---|---|---|---|---|---|---|---|---|---|---|---|---|---|---|---|
| Virus[b] | Strain | AV 93 10135 | AV 93 10040 | AV 96 010149 | AV 96 010151 | AV 98 360019 | AV 96 010154 | AV 98 360020 | AV 98 490013 | TVP-6038 | AV A0 400212 | AV A0 400174 | AV 96 010025 | AV 96 010024 | TAM |
| WWA | AV 9310135 | -- | 100.0 | 86.5 | 86.5 | 86.5 | 85.3 | 85.3 | 82.3 | 82.6 | 79.1 | 79.1 | 83.4 | 85.1 | 71.6 |
| WWA | AV 9310040 | 100.0 | -- | 86.5 | 86.5 | 86.5 | 85.3 | 85.3 | 82.3 | 82.6 | 79.1 | 79.1 | 83.4 | 85.1 | 71.6 |
| WWA | AV 96010149 | 95.3 | 95.3 | -- | 100.0 | 100.0 | 98.1 | 98.5 | 80.1 | 80.3 | 79.5 | 78.8 | 84.4 | 85.5 | 73.7 |
| WWA | AV 96010151 | 95.3 | 95.3 | 100.0 | -- | 100.0 | 98.1 | 98.5 | 80.1 | 80.3 | 79.5 | 78.8 | 84.4 | 85.5 | 73.7 |
| WWA | AV 98360019 | 95.3 | 95.3 | 100.0 | 100.0 | -- | 98.1 | 98.5 | 80.1 | 80.3 | 79.5 | 78.8 | 84.4 | 85.5 | 73.7 |
| WWA | AV 96010154 | 94.8 | 94.8 | 98.8 | 98.8 | 98.8 | -- | 99.6 | 81.3 | 81.5 | 79.3 | 78.6 | 84.4 | 85.1 | 73.2 |
| WWA | AV 98360020 | 95.3 | 95.3 | 99.4 | 99.4 | 99.4 | 99.4 | -- | 81.3 | 81.5 | 79.5 | 78.8 | 84.4 | 85.1 | 73.4 |
| WWA | AV 98490013 | 91.9 | 91.9 | 90.7 | 90.7 | 90.7 | 90.7 | 91.3 | -- | 99.4 | 79.0 | 77.8 | 80.9 | 80.7 | 72.2 |
| WWA | TVP-6038 | 91.9 | 91.9 | 90.7 | 90.7 | 90.7 | 90.7 | 91.3 | 98.8 | -- | 79.1 | 78.0 | 81.1 | 80.1 | 72.4 |
| WWA | AV A0400212 | 88.4 | 88.4 | 88.9 | 88.9 | 88.9 | 88.9 | 89.5 | 86.6 | 86.6 | -- | 95.7 | 77.2 | 77.0 | 69.9 |
| WWA | AV A0400174 | 88.9 | 88.9 | 89.5 | 89.5 | 89.5 | 89.5 | 88.9 | 85.5 | 85.5 | 98.3 | -- | 74.7 | 75.5 | 69.9 |
| WWA | AV 96010025 | 90.7 | 90.7 | 91.3 | 91.3 | 91.3 | 91.3 | 91.3 | 87.8 | 87.8 | 84.9 | 84.9 | -- | 82.8 | 71.6 |
| WWA | AV 96010024 | 93.0 | 93.0 | 94.2 | 94.2 | 94.2 | 94.2 | 94.2 | 88.4 | 88.4 | 86.0 | 86.0 | 89.5 | -- | 72.2 |
| TAM | W-10777 | 77.9 | 77.9 | 78.5 | 78.5 | 78.5 | 78.5 | 78.5 | 78.5 | 78.5 | 80.2 | 80.2 | 77.3 | 78.5 | -- |

[a]Nucleotide and amino acid sequence identities are listed above and below the dashes, respectively.
[b]WWA = Whitewater Arroyo, TAM = Tamiami.

A0400212 from *N. micropus* from the Chaparral Wildlife Management Area) was >95.0%.

The results of previous studies (3,19,20) suggested that the present-day diversity of the arenaviruses is a product of long-term coevolution of the various viruses with their respective principal rodent hosts. In the present study, WWA viral strains AV 9310135 and AV 9310040 (both from *N. albigula*, northwestern New Mexico) appeared to be phylogenetically more closely related to strain AV 96010024 (*N. mexicana*, southeastern Utah) than to strains AV 98490013 and TVP-6038 (both from *N. albigula*, western Oklahoma). This situation suggests that the present-day association of WWA virus with *N. albigula* and *N. mexicana* does not represent a long-term shared evolutionary relationship between virus and rodent species. However, this conclusion assumes that recovery of WWA virus from a rodent represents a principal virus-host relationship. Perhaps some of the virus-positive rodents in the present study were infected by contact with other *Neotoma* species or even non-*Neotoma* rodent species.

The geographic range of the genus *Neotoma* extends from western Canada south to Guatemala, Honduras, and Nicaragua, and includes 33 states in the contiguous United States and 26 of the 32 states in Mexico (21). Thus, if the present-day association of WWA virus with the genus *Neotoma* represents a long-term shared evolutionary relationship between virus and rodent host, the geographic range of the virus may extend far beyond the southwestern United States. WWA virus recently was associated with several human deaths in California (22). Further study is needed to assess the human health significance of this virus in the southwestern United States and other regions in North America in which woodrats are indigenous.

### Acknowledgments

Robert Baker and Terry Yates provided the tissue specimens that were tested for infectious arenavirus; Wen Li Kang amplified and cloned the PCR products generated from isolates AV 96010149, AV 96010151, AV 96010025, and AV 96010024.

This research was supported by the National Institutes of Health grant AI-41435 ("Ecology of emerging arenaviruses in the southwestern United States").

Dr. Fulhorst is assistant professor and member of the World Health Organization Collaborating Center for Tropical Diseases, University of Texas Medical Branch, Galveston. His research interests include the epidemiology of the arenaviruses, hantaviruses, and other viral zoonoses.

### References

1. Southern PJ. *Arenaviridae*: the viruses and their replication. In: Fields BN, Knipe DM, Howley PM, Chanock RM, Melnick JL, Monath TP, et al., editors. Fields virology. 3rd ed. Philadelphia: Lippincott-Raven Publishers; 1996. p. 1505-19.
2. Bowen MD, Peters CJ, Nichol ST. The phylogeny of New World (Tacaribe complex) arenaviruses. Virology 1996;219:285-90.
3. Bowen MD, Peters CJ, Nichol ST. Phylogenetic analysis of the *Arenaviridae*: patterns of virus evolution and evidence for cospeciation between arenaviruses and their rodent hosts. Mol Phylogenet Evol 1997;8:301-16.
4. Weaver SC, Salas RA, de Manzione N, Fulhorst CF, Duno G, Utrera A, et al. Guanarito virus (*Arenaviridae*) isolates from endemic and outlying localities in Venezuela: sequence comparisons among and within strains isolated from Venezuelan hemorrhagic fever patients and rodents. Virology 2000;266:189-95.
5. Jahrling PB, Peters CJ. Lymphocytic choriomeningitis virus. A neglected pathogen of man [editorial]. Arch Pathol Lab Med 1992;116:486-8.
6. Barton LL, Budd SC, Morfitt WS, Peters CJ, Ksiazek TG, Schindler RF, et al. Congenital lymphocytic choriomeningitis virus infection in twins. Pediatr Infect Dis J 1993;12:942-6.
7. Peters CJ, Buchmeier M, Rollin PE, Ksiazek TG. Arenaviruses. In: Fields BN, Knipe DM, Howley PM, Chanock RM, Melnick JL, Monath TP, et al., editors. Fields virology. 3rd ed. Philadelphia: Lippincott-Raven Publishers; 1996. p. 1521-51.
8. Childs JE, Peters CJ. Ecology and epidemiology of arenaviruses and their hosts. In: Salvato MS, editor. The Arenaviridae. New York: Plenum Press; 1993. p. 331-84.
9. Berge T, editor. International catalogue of arboviruses and certain other viruses of the world (supplement). Tamiami (TAM), strain W-10777. Am J Trop Med Hyg 1970;19:1157-8.
10. Calisher CH, Tzianabos T, Lord RD, Coleman PH. Tamiami virus, a new member of the Tacaribe group. Am J Trop Med Hyg 1970;19:520-6.
11. Jennings WL, Lewis AL, Sather GE, Pierce LV, Bond JO. Tamiami virus in the Tampa Bay area. Am J Trop Med Hyg 1970;19:527-36.
12. Fulhorst CF, Bowen MD, Ksiazek TG, Rollin PE, Nichol ST, Kosoy MY, et al. Isolation and characterization of Whitewater Arroyo virus, a novel North American arenavirus. Virology 1996;224:114-20.
13. Kosoy MY, Elliot LH, Ksiazek TG, Fulhorst CF, Rollin PE, Childs JE, et al. Prevalence of antibodies to arenaviruses in rodents from the southern and western United States: evidence for an arenavirus associated with the genus *Neotoma*. Am J Trop Med Hyg 1996;54:570-5.
14. Gonzalez JP, Sanchez A, Ricco-Hesse R. Molecular phylogeny of Guanarito virus, an emerging arenavirus affecting humans. Am J Trop Med Hyg 1995;53:1-6.
15. Thompson JD, Higgins DG, Gibson TJ. CLUSTAL W: improving the sensitivity of progressive multiple sequence alignment through sequence weighting, position-specific gap penalties and weight matrix choices. Nucleic Acids Res 1994;22:4673-80.
16. Weiller GF. TransAlign, version 1.0. Canberra, Australia: Research School of Biological Sciences; 1999.
17. Kumar S, Tamura K, Nei M. MEGA: Molecular Evolutionary Genetics Analysis package, version 1.02. University Park (PA): Pennsylvania State University; 1993.
18. Felsentein J. Confidence limits on phylogenies: an approach using the bootstrap. Evolution 1985;39:783-91.
19. Gonzalez JP, Georges AJ, Kiley MP, Meunier DM, Peters CJ, McCormick JB. Evolutionary biology of a Lassa virus complex. Med Microbiol Immunol 1986;175:157-9.
20. Johnson KM, Webb PA, Justines G. Biology of Tacaribe-complex viruses. In: Lehman-Grube F, editor. Lymphocytic choriomeningitis virus and other arenaviruses. Berlin: Springer-Verlag; 1973. p. 241-58.
21. Musser GG, Carleton MD. Family Muridae. In: Wilson DE, Reeder DM, editors. Mammal species of the world. A taxonomic and geographic reference. Washington: Smithsonian Institution Press; 1993. p. 501-755.
22. Centers for Disease Control and Prevention. Fatal illnesses associated with a New World arenavirus-California, 1999-2000. MMWR Morb Mortal Wkly Rep 2000;49:709-11.
